# Metformin treatment of diverse *Caenorhabditis* species reveals the importance of genetic background in longevity and healthspan extension outcomes

**DOI:** 10.1111/acel.13488

**Published:** 2021-11-27

**Authors:** Brian Onken, Christine A. Sedore, Anna L. Coleman‐Hulbert, David Hall, Erik Johnson, Eleanor Grace Jones, Stephen A. Banse, Phu Huynh, Suzhen Guo, Jian Xue, Esteban Chen, Girish Harinath, Anna C. Foulger, Elizabeth A. Chao, June Hope, Dipa Bhaumik, Todd Plummer, Delaney Inman, Mackenzie Morshead, Max Guo, Gordon J. Lithgow, Patrick C. Phillips, Monica Driscoll

**Affiliations:** ^1^ Nelson Biological Laboratories Department of Molecular Biology and Biochemistry Rutgers University Piscataway New Jersey USA; ^2^ Institute of Ecology and Evolution University of Oregon Eugene Oregon USA; ^3^ The Buck Institute for Research on Aging Novato California USA; ^4^ Division of Aging Biology National Institute on Aging Bethesda Maryland USA

**Keywords:** anti‐diabetes drug, biguanide, genetic diversity, healthspan, longevity

## Abstract

Metformin, the most commonly prescribed anti‐diabetes medication, has multiple reported health benefits, including lowering the risks of cardiovascular disease and cancer, improving cognitive function with age, extending survival in diabetic patients, and, in several animal models, promoting youthful physiology and lifespan. Due to its longevity and health effects, metformin is now the focus of the first proposed clinical trial of an anti‐aging drug—the Targeting Aging with Metformin (TAME) program. Genetic variation will likely influence outcomes when studying metformin health effects in human populations. To test for metformin impact in diverse genetic backgrounds, we measured lifespan and healthspan effects of metformin treatment in three *Caenorhabditis* species representing genetic variability greater than that between mice and humans. We show that metformin increases median survival in three *C*. *elegans* strains, but not in *C*. *briggsae* and *C*. *tropicalis* strains. In *C*. *briggsae*, metformin either has no impact on survival or decreases lifespan. In *C*. *tropicalis*, metformin decreases median survival in a dose‐dependent manner. We show that metformin prolongs the period of youthful vigor in all *C*. *elegans* strains and in two *C*. *briggsae* strains, but that metformin has a negative impact on the locomotion of *C*. *tropicalis* strains. Our data demonstrate that metformin can be a robust promoter of healthy aging across different genetic backgrounds, but that genetic variation can determine whether metformin has positive, neutral, or negative lifespan/healthspan impact. These results underscore the importance of tailoring treatment to individuals when testing for metformin health benefits in diverse human populations.

## Graphical abstract

We monitored metformin impact in nine strains spanning three Caenorhabditis species that feature a nucleotide diversity greater than that between mouse and humans. We find that metformin promotes healthy aging across diverse genetic backgrounds, but that genetic background can determine whether metformin has a positive, neutral, or negative effect. Data demonstrate the potential broad reach of metformin but also underscore that individual genetics will underlie efficacy over a diverse test set.

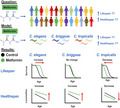

## 1 INTRODUCTION

A major goal of current aging research is to identify strategies to extend youthful physiology without increasing the time spent in decrepit old age. As the prevalence of diseases associated with the highest rates of mortality—heart disease, cancer, Alzheimer's disease, and type 2 diabetes—increases with age, interventions that confer anti‐aging outcomes are anticipated to also lower age‐associated disease incidence. One approach toward enhancing older age health is to identify small molecule interventions that promote healthy aging. Still, as with nearly all pharmaceuticals (Dugger et al., 2018), individual genetic differences might be expected to lead to differential responses to longevity‐enhancing compounds. Indeed, evaluation of candidate medicines without recognition of the underlying diversity in both disease causation and drug response in the patient population has been suggested as a key contributor to drug development failures (Ma & Zemmel, 2002).

Metformin, a biguanide derived from the French lilac *Galega officinalis*, has received considerable attention as a compound with potential to promote healthy aging (Glossmann & Lutz, 2019; Novelle et al., 2016). Metformin is the most widely prescribed anti‐diabetic medication, effective in reducing blood sugar levels with minimal side effects (Glossmann & Lutz, 2019; Novelle et al., 2016). In addition to its anti‐diabetic effects, metformin has also been found to confer beneficial effects in the treatment of cardiovascular disease and some forms of cancer, and to improve cognition in older adults (Novelle et al., 2016; Pryor & Cabreiro, 2015; Valencia et al., 2017). Adding to the considerable interest in metformin benefits, there is strong evidence that metformin can extend longevity and promote healthy aging in multiple model organisms (Barzilai et al., 2016; Novelle et al., 2016; Pryor & Cabreiro, 2015), and is associated with extended survival in type 2 diabetic patients compared with matched healthy controls (Pryor & Cabreiro, 2015; Valencia et al., 2017). The documented broad benefits of metformin in diabetic and cancer patients have anchored the premise for the first clinical trial of a healthspan‐promoting compound (the Targeting Aging with Metformin—TAME) (Barzilai et al., 2016).

Although the anti‐hyperglycemic effects of metformin are well documented, the complex mechanism(s) of metformin action are not fully understood. Metformin has a significant impact on metabolic machinery, with targets including mitochondrial complex I, the cellular energy sensor AMPK, and mTOR (Pryor & Cabreiro, 2015). Different metformin doses can engage different molecular processes. Increasing the complexity of metformin's interface with physiology, interactions of metformin with the microbiome can also have profound health consequences in the nematode *Caenorhabditis elegans*, *Drosophila melanogaster* and vertebrates, including humans (Pryor & Cabreiro, 2015). The picture that emerges is that the multiple levels at which metformin engages physiology may elicit a range of individual responses within a population.

Simple animal models have been extensively studied to yield insight into fundamental biological processes, including conserved responses to pharmacological treatments, although such studies are frequently conducted using a single strain or genetic background. The *Caenorhabditis* Intervention Testing Program (CITP) explicitly addresses this common limitation within longevity studies by testing potential lifespan‐ and healthspan‐promoting interventions across a broad range of genetic backgrounds, exploiting the diversity of the *Caenorhabditis* genus by using strains from three species—*C*. *elegans*, *C*.* briggsae*, and *C*. *tropicalis*—that represent millions of years of divergent evolution. In addition, the CITP emphasizes experimental reproducibility by conducting studies at three independent sites using identical standard operating procedures, striving toward minimal lab‐to‐lab variation in results but always evaluating sources of variation as a critical component of data analysis. In addition to lifespan evaluation, the CITP makes use of a relatively easy‐to‐implement healthspan assay that delivers multi‐parameter scores on nematode locomotory ability with age. These lifespan and mobility health analyses provide reproducible data on the efficacy of anti‐aging interventions.

How metformin treatment plays out for promoting general health in a genetically diverse human population is clearly of great interest in current gerontology. Here we report on the impact of metformin on longevity and old age mobility measures in a genetically diverse test group of *Caenorhabditis* species. We find that metformin increases the median lifespan of all tested *C*. *elegans* strains, but not the median lifespan of strains from *C*. *briggsae* or *C*. *tropicalis*. Metformin did not improve *C*. *briggsae* survival at any dose. In *C*. *tropicalis* strains, increasing metformin concentrations decrease lifespan in a dose‐dependent manner. Notably, analysis of locomotory healthspan revealed positive outcomes of metformin treatment in all *C*. *elegans* strains and in two of the three *C*. *briggsae* strains, highlighting a “quality of life” mobility benefit distinct from life extension outcomes for some strains. For *C*. *tropicalis* strains, locomotion was generally more impaired consequent to metformin treatment. Our data exhibit low variation in lifespan and healthspan results across the three CITP test sites, meeting the CITP goal of generating highly reproducible results.

Overall, we demonstrate that metformin stands out as an effective pro‐healthspan compound in a genetically diverse test set of *Caenorhabditis*. At the same time, our findings highlight that some genetic variants can either be partially refractory to metformin benefits or can suffer deleterious outcomes of treatment. Our data therefore raise the potential need for attention to individualized medicine considerations in the drive toward dissecting, and administering, the benefits of metformin in the human population.

## 2 RESULTS

### 2.1 Metformin increases median lifespan in three *C*.* elegans* Strains

The *Caenorhabditis* Intervention Testing Program (CITP) is a collaborative effort conducted at three geographically separated research labs in which we seek to identify compounds that confer reproducible longevity extension and health benefits to a genetically diverse panel of *Caenorhabditis* strains. The underlying premise of this consortium is that compounds that confer robust outcomes across a genetically heterogeneous population should have an enhanced probability of engaging conserved biochemical pathways that promote healthy aging across phyla. The CITP effort relies on purchase of common reagents, adherence to highly detailed protocols, and frequent and regular communication (Lithgow et al., 2017). We used this rigorous framework to conduct manual survival assays in which each of the three labs conducted three biological repeats at multiple metformin concentrations. Our work focused on three strains from *C*. *elegans*, three strains from *C*. *briggsae*, and three strains from *C*. *tropicalis*, originally selected to maximize genetic and geographic diversity within each species (Kiontke et al., 2011; Kiontke & Fitch, 2005). Overall, comparisons of genome sequences and specific gene sequences indicate that the genetic difference in this test set is comparable in range to that which results from sampling genomes from mice to humans (Kiontke et al., 2011; Kiontke & Fitch, 2005).

For *C*. *elegans*, we studied the canonical lab wild type strain N2 along with MY16 and JU775 isolates, among which there is an average per site nucleotide difference of around 0.2% (see Methods), comparable to that within the human population (C. et al., 2015; Nam et al., 2017; Sachidanandam et al., 2001). We analyzed ~24,600 total individual survival outcomes at multiple metformin concentrations, including one previously documented to increase *C*. *elegans* N2 lifespan (50 mM) (Pryor & Cabreiro, 2015). Note that the *C*. *elegans* collagenous cuticle and the reinforced gut structure often necessitate use of much higher compound concentrations than might be required in a culture model (Holden‐Dye & Walker, 2014; Rand & Johnson, 1995) and the high concentrations applied to plate media likely do not reflect the physiological levels within treated animals.

**FIGURE 1:**
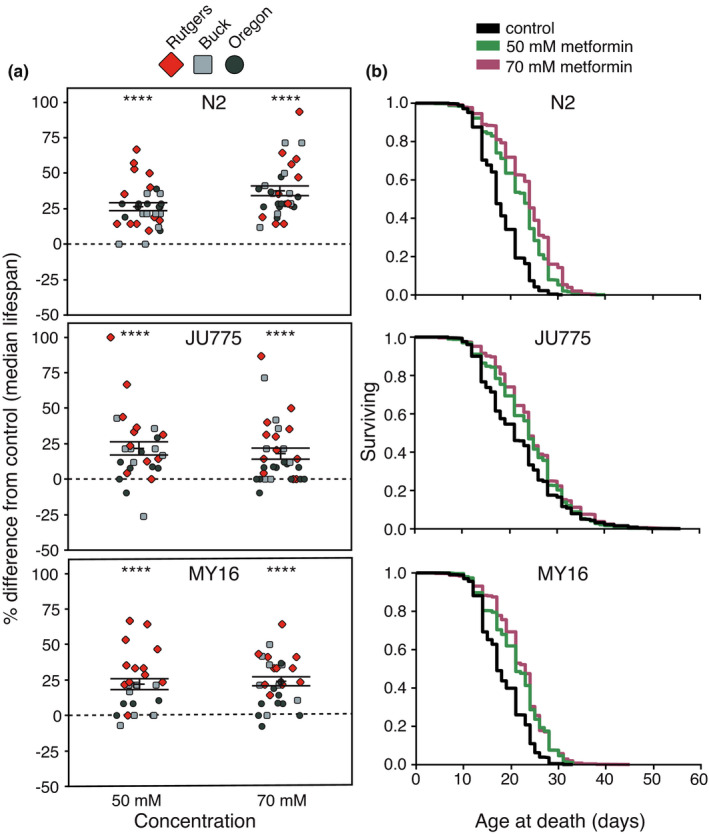
Adult exposure to metformin extends the median lifespan of three *C*. *elegans* strains (N2, JU775, MY16) at 50 mM or 70 mM metformin. (a) Each point represents the change in median lifespan from an individual trial plate (compound treated) relative to the specific control (vehicle only) conducted. The bars represent the mean +/− the standard error of the mean. Replicates were completed at the three CITP testing sites (blue square Buck Institute, red diamond Rutgers, dark green circle Oregon). (b) Kaplan–Meier survival curves of *C*. *elegans* strain N2, JU775, and MY16 during adult exposure to metformin (black control, teal 50 mM, blush 70 mM). Asterisks represent *p*‐values from the Cox Proportional Hazards (CPH) model such that ****p < 0.0001, ***p < 0.001, **p < 0.01, and *p < 0.05 (Datasets S2 and S3). All lifespan curves represent averages pooled across all data; see Figure S1 for details on individual lifespan curves

Our results confirm pro‐longevity effects in *C*. *elegans* reference strain N2, with significant increases in median lifespan at two metformin concentrations (Figure 1a; 50 mM, a 35% increase; 70 mM, a 41% increase; *p* < 0.001 for both; see Figure S1 and Datasets S2 and S3 for summary of all lifespan data). *C*. *elegans* strains JU775 and MY16 also exhibit positive effects on median lifespan with metformin treatment (Figure 1a; JU775 shows a 14% increase for both 50 mM and 70 mM; MY16 on 50 mM, a 23% increase, and, on 70 mM, a 35% increase; *p* < 0.001 for both). These median survival benefits are also reflected in right‐shifted survival curves for metformin treatment in all three strains (Figure 1b; *p* < 0.001 for all). Maximal lifespan is also increased with 50 and 70 mM metformin treatment in all three strains (calculated as age at 90% population death; see Figure S2). Together, our results show that metformin increases lifespan in a well‐characterized test set of genetically diverse *C*. *elegans* strains that model the diversity found within the human population, demonstrating the robust capacity for this biguanide to confer adult survival benefits.

### 2.2 The longevity extension effects of metformin are not observed in *C*.* briggsae* and *C*.* tropicalis* species

**FIGURE 2:**
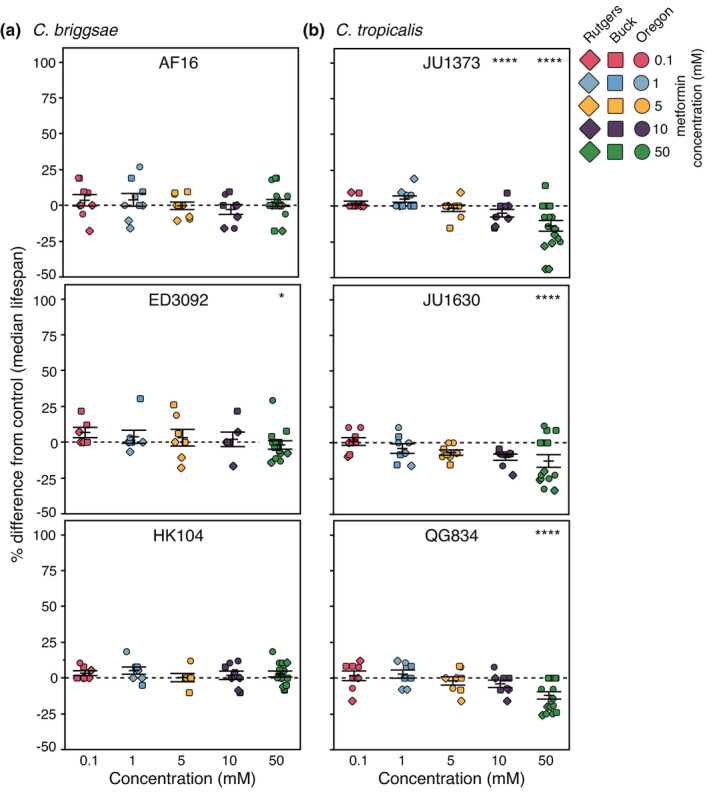
Adult exposure to metformin does not extend median lifespan in two *Caenorhabditis* species at any concentration tested. A dose response of the change in median lifespan during adult exposure to metformin is shown for (a) three *C*. *briggsae* strains (AF16, ED3092, HK104), and (b) three *C*. *tropicalis* strains (JU1373, JU1630, QG834). Each point represents the change in median lifespan from an individual trial plate (compound treated) relative to the specific control (vehicle only) conducted. The bars represent the mean +/− the standard error of the mean. Colors correspond to the concentrations tested (salmon 0.1 mM, light blue 1 mM, orange 5 mM, purple 10 mM, and teal 50 mM). Replicates were completed at the three CITP testing sites (square Buck Institute, diamond Rutgers, circle Oregon). Asterisks represent *p*‐values from the CPH model such that ****p < 0.0001, ***p < 0.001, **p < 0.01, and *p < 0.05

**FIGURE 3:**
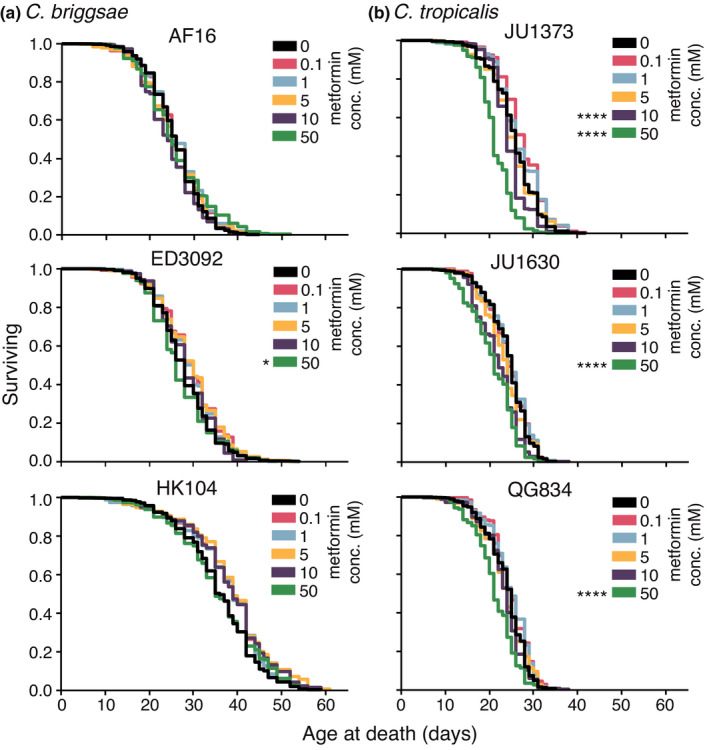
Adult exposure to metformin does not extend lifespan in *C*. *briggsae* or *C*. *tropicalis* strains at any concentration tested. Kaplan–Meier survival curves are shown for survival during adult exposure to various concentrations of metformin for (a) three *C*. *briggsae* strains (AF16, ED3092, HK104), and (b) three *C*. *tropicalis* strains (JU1373, JU1630, QG834). Replicates were completed at the three CITP testing sites. Asterisks represent *p*‐values from the CPH model such that ****p < 0.0001, ***p < 0.001, **p < 0.01, and *p < 0.05 (details in Supplemental Datasets S2 and S3). Lifespan curves represent pooled averages across all experiments; see Figure S1 for details on individual lifespan curves

To test how broadly within the *Caenorhabditis* genus metformin impact on longevity could be detected, we conducted survival analyses on *C*. *briggsae* strains AF16, ED3092, and HK104. (100 million years of separation between *C*. *elegans* and *C*. *briggsae* (Coghlan & Wolfe, 2002)). Metformin did not significantly increase median lifespan in *C*. *briggsae* over a broad range of tested doses (0.1–50.0 mM; Figure 2a; at the 50 mM dose, metformin significantly decreases ED3092 median lifespan, *p* < 0.05), nor did metformin increase survival of *C*. *briggsae* strains at any concentration (Figure 3a; similar to our median survival results, the 50 mM dose significantly shortened the survival curve of ED3092 animals, *p* < 0.05). Because our previous studies identified compounds that were efficacious in both *C*. *elegans* and *C*. *briggsae* (Lucanic et al., 2017), lack of response appears compound‐specific rather than being attributable to a general challenge with compound interventions in *C*. *briggsae*.

We also treated *C*. *tropicalis* strains JU1373, JU1630, and QG834 with metformin and assayed survival. For *C*. *tropicalis* strains, we observed dose‐dependent reductions in median lifespan, with the highest doses conferring clear detrimental effects (Figure 2b; in JU1373, 10 and 50 mM metformin decrease median lifespan by 12% and 16%, respectively; *p* < 0.001 for both; in JU1630, 50 mM metformin decreases median lifespan by 19%, *p* < 0.001; in QG834, 50 mM metformin decreases median lifespan by 16%, *p* < 0.001). Survival curves for the range of tested metformin concentrations on *C*. *tropicalis* parallel the median lifespan data: *C*. *tropicalis* survival is shortened in a dose‐dependent manner (Figure 3b). In all, our data highlight a dramatic range of metformin responses among representative members of three *Caenorhabditis* species: metformin can increase lifespan across *C*. *elegans* strains, is neutral in *C*. *briggsae* strains, and is deleterious in *C*. *tropicalis* strains.

### 2.3 Metformin promotes locomotory maintenance in an expanded genetic diversity test set

Mobility decline is a nearly universal feature of aging, and mobility measures are widely used to assess the physiological age of older patients (Soubra et al., 2019). In *C*. *elegans*, a progressive decline in swim vigor is an often‐measured indicator of aging, and thus we analyzed features of swimming as a measure of locomotory ability likely to reflect neuromuscular health (Laranjeiro et al., 2017, 2019). We used CeleST (*C*. *elegans* Swim Test) computer vision analysis software for collection and analysis (Restif et al., 2014). CeleST scores eight different parameters of locomotion and body posture (see the Supplementary Information for details on the CeleST parameters). We weighted the eight measurement values to generate a composite swimming score meant to reflect overall vigor (https://doi.org/10.6084/m9.figshare.c.5126579) which we use, reminiscent of the human frailty index (Palliyaguru et al., 2019), as a biomarker of health and aging quality. Although locomotory decline occurs during adult life for all *Caenorhabditis* strains, timing of maximal decline rate differs somewhat among *C*. *elegans*, *C*. *briggsae* and *C*. *tropicalis*; therefore, we scored on adult day 6 and 12 for *C*. *elegans* and *C*. *tropicalis* and adult day 8 and 16 for *C*. *briggsae* in an effort to analyze at timepoints reflecting similar features on the decline slope (*C*. *briggsae* strains tend to be longer lived than *C*. *elegans* and *C*. *tropicalis* strains; steepest decline tends to be more evident in chronologically older *C*. *briggsae* animals).

**FIGURE 4:**
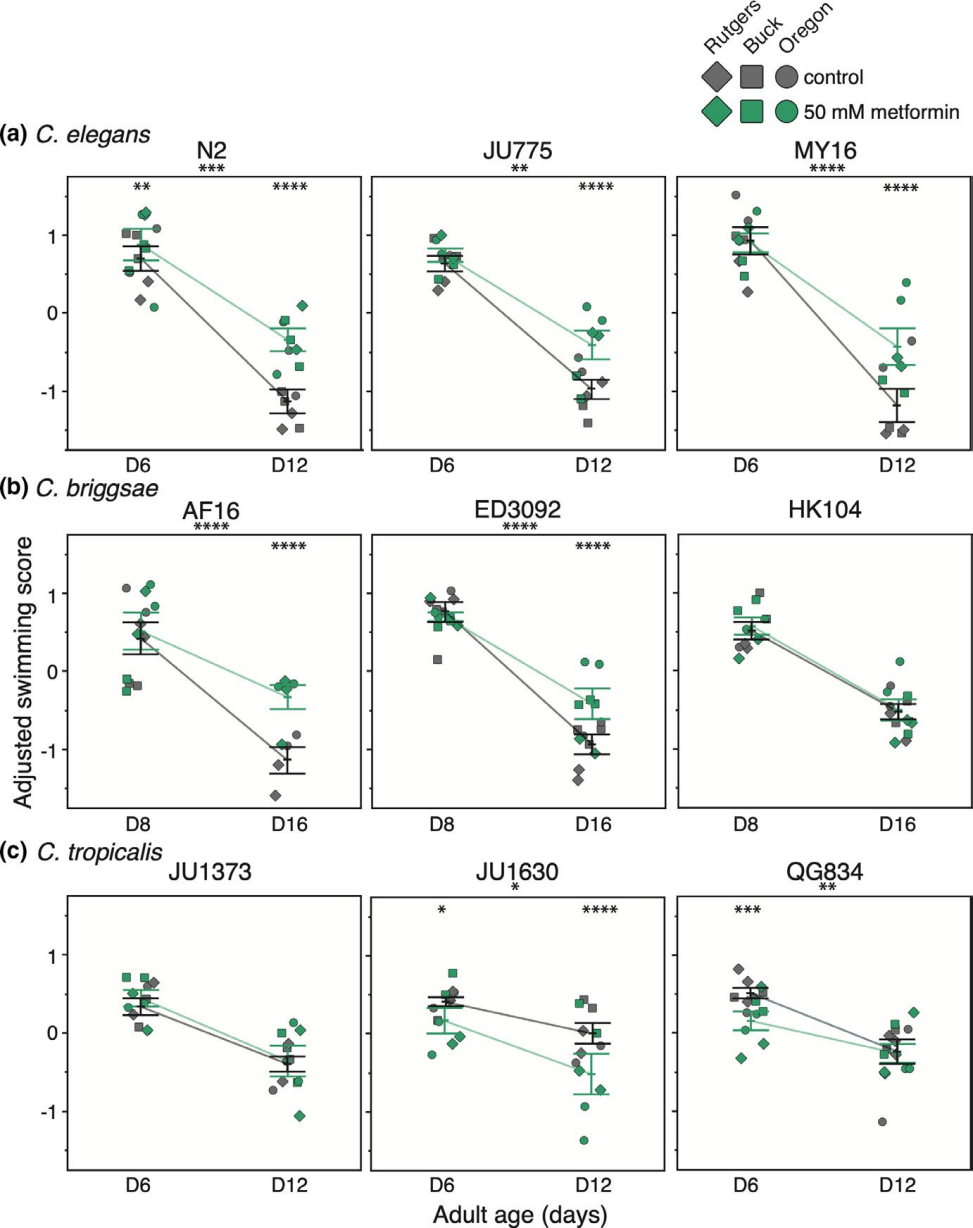
Metformin slows the age‐related decline in swimming ability in multiple, but not all, *Caenorhabditis* species and strains. The mean adjusted swimming score is shown for (a) three *C*. *elegans* strains (N2, JU775, MY16), (b) three *C*. *briggsae* strains (AF16, ED3092, HK104), and (c) three *C*. *tropicalis* strains (JU1373, JU1630, QG834) tested at two ages (adult days 6 and 12 for *C*. *elegans* and *C*. *tropicalis*, adult days 8 and 16 for *C*.* briggsae*). Each point represents the mean from one trial, the bars represent the mean +/− the standard error of the mean, and the colors correspond to the treatment conditions starting at day 1 of adulthood (gray control, teal 50 mM metformin). Replicates were completed at the three CITP testing sites (square Buck Institute, diamond Rutgers, circle Oregon). Asterisks outside each plot represent *p*‐values from the Type‐III ANOVA and indicate a significant age‐by‐compound interaction for that strain, while asterisks inside each plot represent *p*‐values from the linear mixed model such that ****p < 0.0001, ***p < 0.001, **p < 0.01, and *p < 0.05 (Details in Dataset S1)

Aging *C*. *elegans* strains treated with 50 mM metformin exhibit increased mean composite swimming scores (Figure 4a; in N2, metformin significantly increases the mean composite swimming score on adult day 6 and 12 of adulthood; *p* < 0.01 and *p* < 0.0001, respectively; in JU775 and MY16, metformin increases the composite swimming score on adult day 12; *p* < 0.0001 for both). Thus, metformin treatment extends both longevity and swim vigor in aging *C*. *elegans* strains.

Although metformin treatment had no positive impact on the longevity of any tested *C*. *briggsae* strain (Figures 2a and 3a), we recorded significant increases in locomotory ability with 50 mM metformin treatment in *C*. *briggsae* strains AF16 and ED3092 (Figure 4b; both strains show increased mean composite swim scores on adult day 16 of life; *p* < 0.0001). *C*. *briggsae* strain HK104 appears to be refractory to metformin as assessed by swim measures. Still, the important point is that although metformin does not extend longevity in *C*. *briggsae* strains AF16 and ED3092 (and actually decreases lifespan at 50 mM in ED3092; Figures 2a and 3a), benefits to locomotory capacity in these strains are clear. We conclude that, in some genetic backgrounds, metformin can confer older age health benefits in the absence of impacts on longevity, and we note a beneficial impact on movement vigor across species that diverged approximately 100 million years ago (Coghlan & Wolfe, 2002).

By contrast, 50 mM metformin conferred detrimental effects in two of three *C*. *tropicalis* strains. Metformin‐treated strain JU1630 exhibits decreased swimming ability on adult days 6 and 12 (*p* < 0.05 and *p* < 0.0001, respectively); likewise metformin‐treated strain QG834 exhibits decreased swim ability on adult day 6 (*p* < 0.001) compared to untreated controls (Figure 4c; we note that there is a significant interaction effect present in QG834 (*p* < 0.01), with a diminished slope between the day 6 and day 12 timepoints vs. controls). *C*. *tropicalis* strain JU1373 did not exhibit a change in swimming ability with metformin treatment on either day of measurement, although we note that JU1373 is generally a more vigorous swimmer than the other tested *C*. *tropicalis* strains such that detrimental effects with metformin might be evident in older JU1373 nematodes when swimming ability declines to typical JU1630 and QG834 levels. We conclude that metformin can have a negative impact on some *C*. *tropicalis* strains. We also note that the three *C*. *tropicalis* strains and *C*. *briggsae* HK104 show the slowest rates of locomotory decline of all tested strains (Figure 4); it is possible that additional metformin health effects could be revealed by performing the CeleST assay in older animals (for technical and practical reasons, we did not include late‐life timepoints in this study).

### 2.4 Metformin stands out as an anti‐diabetic compound that promotes healthy aging

As metformin demonstrates a striking ability to promote healthy aging across a range of genetic backgrounds, we asked whether other anti‐diabetic compounds might have the capacity to confer similar outcomes. We tested six type 2 diabetes medications (bromocriptine, dapagliflozin, sitagliptin, nateglinide, pioglitazone, and glipizide) that lower blood sugar levels via a variety of mechanisms for longevity effects in representatives of the three *Caenorhabditis* species (Figure S3). Of the six compounds, only one, bromocriptine, a dopamine agonist thought to act as an anti‐diabetic by inhibiting increased plasma glucose levels associated with abnormally elevated hypothalamic activity, had a substantial longevity impact (details in Figure S3A). Although this mini screen was limited in scope and depth, we can conclude that only particular anti‐diabetes interventions confer longevity and health benefits in *Caenorhabditis* test models.

### 2.5 Little or no variability in longevity and locomotory data among labs

Our previous longevity studies of 22 natural isolates in *C*. *elegans*, *C*. *briggsae*, and *C*. *tropicalis* species indicated high reproducibility of data across the three CITP labs (Lucanic et al., 2017), a key objective of our initiative. Tables S1 and S2 show longevity and locomotory data from our metformin analyses partitioned into different potential sources of variation using a general linearized model. Our results are reproducible: for the 50 mM metformin longevity studies, there was little variation among labs (5.1%), and the among‐lab variation for our CeleST locomotory data was similarly low (4.5%). These percentages are even lower than what we found previously, where the variation for longevity data among labs was 7.5% (Lucanic et al., 2017). Overall, our data demonstrate reproducibility in metformin outcomes among tightly matched studies.

## 3 DISCUSSION

The anti‐diabetes drug metformin is under intensive study as a general health‐promoting treatment, with human trials planned to investigate potential benefits to health maintenance in the elderly (Barzilai et al., 2016). Critical for determining benefits across the diverse human population is an assessment of metformin outcomes in a genetically rich population, which is the key question that we address here in *Caenorhabditis* models. We examined the efficacy of metformin in promoting lifespan and locomotory health in large populations from diverse *Caenorhabditis* genetic backgrounds. Our data confirm that metformin can increase lifespan of the canonical lab *C*. *elegans* reference strain N2 (Pryor & Cabreiro, 2015), and show that metformin can confer pro‐longevity and mobility maintenance effects in two additional *C*. *elegans* strains. Data thus demonstrate a remarkable capacity for metformin to increase lifespan and improve physical maintenance across diverse genetic backgrounds within the *elegans* species. At the same time, analysis of metformin impact in closely related species revealed that genetic background can play a significant role in outcome. Regarding longevity, we saw no change in *C*. *briggsae* lifespan with metformin exposure, with the exception of a single strain that showed decreased survival on the highest tested dose of metformin, while *C*. *tropicalis* strains show decreased median lifespan with increasing metformin concentrations. We found that metformin improved locomotory healthspan in two out of the three tested *C*. *briggsae* strains but in *C*. *tropicalis* strains, metformin decreases mobility outcomes, similar to *C*. *tropicalis* lifespan results. We discuss details, caveats and implications below.

### 3.1 The *Caenorhabditis* intervention testing program as a focal point for advancing testing of interventions for mammalian anti‐aging therapeutics

The goal of the CITP effort is to use rigorous protocols (identical techniques/materials across test sites; please see Lithgow et al. (2017) for details) to provide high‐quality, reproducible data on small molecule/plant extract effects on lifespan and healthspan in a genetically diverse test set from the *Caenorhabditis* genus. A core underlying hypothesis is that interventions that are bioactive against a diverse population most likely target conserved longevity/health pathways, and therefore may have an enhanced chance for success in mammalian testing.

The three different *Caenorhabditis* species used by CITP—*C*. *elegans*, *C*. *briggsae*, and *C*. *tropicalis*—were selected in part because these are hermaphroditic species, facilitating ease of culture in the lab. The features/special considerations of the hermaphroditic *Caenorhabditis* genomes have been documented (Hill et al., 2006; Woodruff et al., 2010), and sequences for many wild *Caenorhabditis* strains are available (Stevens et al., 2019) (CITP is in the process of publishing details of the strain genome comparisons for the strains we used here (MicroPublication Biology, in prep)). In general, the 0.2% per site nucleotide difference among the three *C*. *elegans* strains is similar to genetic variation between mice and humans (Kiontke et al., 2011; Kiontke & Fitch, 2005) and the nine representative strains selected can be considered a plausible genetic diversity representation.

Our previous work using CITP protocols to test potential pro‐longevity compounds showed that 6 out of 12 tested candidate longevity interventions conferred reproducible lifespan‐promoting effects, with one, Thioflavin‐T, impacting all three species (Lucanic et al., 2017). Our current results testing metformin demonstrate strong reproducibility—lifespan and healthspan exhibited low lab‐to‐lab variation (Table S1), similar to what we found in previous studies.

### 3.2 Metformin efficacy exhibits strain‐specific differences

We found species‐specific lifespan/healthspan impact with metformin treatment. Metformin has beneficial effects in all three *C*. *elegans* strains (increased median survival and locomotory ability) and in two *C*. *briggsae* strains (increased locomotory ability only), but is toxic in *C*. *tropicalis* strains. What might account for these differential effects? There are two means for a small molecule to enter nematode tissues—either via ingestion, or through absorption across the cuticle. The efficiency of absorption depends on the lipophilicity of the compound, but it is estimated that, in *C*. *elegans* N2, drug dose should be 1000× greater than the target concentration in tissues (Holden‐Dye & Walker, 2014; Rand & Johnson, 1995). Relatively high metformin doses (50 and 70 mM) are required for beneficial effects in *C*. *elegans* strains, suggesting that metformin, a highly polar molecule, may not readily cross the cuticle. We note that if the 1000× rule is applied, metformin concentrations in nematode tissues in our studies would approximate 50–70 µM, similar to pharmacologic concentrations reported for metformin in mammals (40–70 µM (He & Wondisford, 2015)). That metformin has no effect on the lifespan of any tested *C*. *briggsae* strain at a range of concentrations from 100 μM to 50 mM may reflect differences in cuticle or epitheial boundary makeup between the two species. Since 50 mM metformin improves locomotory ability in two strains, it is evident that *C*. *briggsae* is able to absorb metformin (or its bacterially generated metabolites) at high concentrations, but this healthspan‐promoting dose is not sufficient to increase median lifespan in this species.

Metformin has multiple cell targets and details of drug intersection with these is likely related to uptake capacity, bioactive dose, metabolites, turnover rate, and genetically specified pathway flux levels. In *C*. *elegans* N2, metformin has been shown to disrupt mitochondrial complex I function, increasing ROS levels and leading to decreased mTORC1 kinase activity via the Rag family of GTPases (Pryor & Cabreiro, 2015; Wu et al., 2016). Metformin also activates the AMP‐activated protein kinase (AMPK) energy sensor, and both TOR inhibition and AMPK activation require signaling via the v‐ATPase‐Ragulator lysosomal pathway (Chen et al., 2017). mTORC1 inhibition leads to activation of the SKN‐1/Nrf2 transcription factor and induction of expression of the ACAD10 protein that regulates growth and longevity (Wu et al., 2016). In addition, metformin has been shown to increase lifespan, at least in part, by altering folate and methionine metabolism of the *E*. *coli* food source for nematodes (Pryor & Cabreiro, 2015). Metformin has also been shown to alter the microbiota of humans and flies in a conserved fashion (Pryor et al., 2019), suggesting that metformin may similarly impact microbiomes across species to promote healthy aging, and highlighting the relevance of considering the microbiome when tailoring individual metformin treatment.

Differences in *C*. *briggsae* and *C*. *tropicalis* sensitivity to metformin treatment that we observe may reflect differential regulation of any of the elements in the complex metformin response pathways. During development, gene expression patterns appear to be conserved in *C*. *elegans* and *C*. *briggsae* (Grun et al., 2014; Lu et al., 2020); probing adult transcriptomes and metabolomes for changes with metformin treatment might likely shed light on the differential effects of metformin in the three tested species. We also note that we use FUdR (floxuridine) in our studies to prevent progeny production—it is possible that FUdR treatment in *C*. *briggsae* and *C*. *tropicalis* contributes some of the species‐specific differences we observe with metformin treatment (there appears to be no synthetic interaction between FUdR and metformin, however; see Methods for details).

It is also important to note that, although metformin is far less toxic than other biguanides, high doses of metformin have negative lifespan and healthspan effects in flies, mice, and multiple *C*. *elegans* N2 assays (Pryor & Cabreiro, 2015). AMPK, SKN‐1/Nrf2, and the peroxiredoxin hydrogen peroxide scavenger PRDX‐2 are thought to mediate protective mechanisms for metformin‐induced stresses (Pryor & Cabreiro, 2015). Our data show that metformin has a dose‐dependent detrimental effect on lifespan in *C*. *tropicalis*, and that 50 mM metformin decreases locomotory ability in two *C*. *tropicalis* strains. These results indicate that *C*. *tropicalis* strains can absorb metformin or the *E*. *coli* metabolites associated with metformin, but clearly the consequences of uptake do not activate benefits. While the nature of this toxicity is unknown, one possibility is that the AMPK/SKN‐1 defensive mechanism is less responsive to metformin exposure in *C*. *tropicalis* versus *C*. *elegans*. Genetic differences to be identified in the future likely underlie the different responses to metformin between *C*. *elegans* and *C*. *tropicalis*.

It is clear that deciphering the mechanistic basis of different strain responses to metformin could inform on biology of high relevance to understanding outcome efficacy in higher organisms. The mandate of the CITP, however, is highly focused on the testing and identification of pharmacological interventions that reproducibly promote lifespan and healthspan benefits across a genetically diverse test set. Thus while mechanistic studies (even by a single lab within the CITP) would likely reveal important details on how metformin impacts the different *Caenorhabditis* strains, the CITP effort focuses on reasonable throughput testing of compounds through a reproducible longevity/mobility test protocol. The intended integration of CITP outcome in the field is that robust CITP findings should inspire outside mechanistic studies on uptake, transcription, metabolism, and pathway biochemistry that extend medically relevant understanding of different metformin treatment outcomes in genetically diverse populations.

### 3.3 Metformin treatment can decouple longevity and mobility health

Our data show a strong correlation between metformin impact on longevity and locomotory prowess in different *C*. *elegans* strains, consistent with previous reports on the beneficial effects of metformin on both lifespan and healthspan in *C*. *elegans* N2 (Pryor & Cabreiro, 2015). In two of three tested *C*. *briggsae* strains, metformin can promote locomotory health benefits without impacting either median survival or maximal lifespan. These observations speak to the fundamental question as to whether interventions can be identified that extend the period of healthy functionality without a major impact on longevity (Bansal et al., 2015). That metformin may increase the maintenance of locomotory health without extending longevity has been suggested for *C*. *elegans* (Onken & Driscoll, 2010) and is demonstrated here for *C*. *briggsae*. A maintenance‐promoting intervention outcome should be a sought after outcome in human trials.

### 3.4 Implications for human metformin studies

Our data confirm previous reports that metformin promotes lifespan and healthspan in *C*. *elegans* N2 (Pryor & Cabreiro, 2015) and show that these benefits can extend to a representative, genetically diverse set of *C*. *elegans* strains. At the same time, outcomes do not fully extend to closely related *Caenorhabditis* species. Metformin increases lifespan in crickets, but not in the fruit fly model *Drosophila*, and metformin's lifespan/healthspan effects in rodents are mixed (Pryor & Cabreiro, 2015). The Strong et al. report, in which no lifespan benefits were seen with metformin treatment in a population of genetically heterogeneous mice, is particularly notable, as this study was performed by the NIA Intervention Testing Program using fundamental assay design principles upon which the CITP is based (parallel testing of compounds at three independent sites) (Strong et al., 2016). In humans, clinical trials in populations with type 2 diabetes show that metformin increases overall survival, lowers incidence of cancer and cardiovascular disease, and improves cognitive function (Novelle et al., 2016; Pryor & Cabreiro, 2015; Valencia et al., 2017), although there is of yet no evidence that metformin can promote health or increase lifespan in healthy populations. In a recent perspective focused on the TAME proposal in which metformin would be the first anti‐aging intervention to be studied in a clinical trial (Barzilai et al., 2016), Konopka and Miller point out that since the trial would look to see if metformin can delay the onset of new age‐related conditions in individuals already beset with chronic disease, the study design would not constitute a test of metformin capacity to extend the amount of time a healthy individual maintains a healthy state (“healthspan”, *per se* (Kaeberlein, 2018; Konopka & Miller, 2019)). Our data highlight the value of locomotory healthspan as an indicator of metformin efficacy—while locomotory capacity tracks well with lifespan in *C*. *elegans* strains, we see only health benefits with metformin treatment in *C*. *briggsae*, suggesting that metformin impact on the quality of locomotory aging might be greater, or at least more apparent, than metformin longevity effects across diverse genetic backgrounds. We suggest that measuring healthspan metrics in healthy populations in metformin trials is essential, and that these data may reveal metformin benefits that might otherwise be overlooked.

We also identify dose as being particularly important in metformin studies in heterogeneous genetic backgrounds—50 mM metformin, which is beneficial in *C*. *elegans* and *C*. *briggsae*, is toxic in *C*. *tropicalis*. Previous studies where metformin failed to increase lifespan/healthspan in flies, mice, and rats (Martin‐Montalvo et al., 2013; Slack et al., 2012; Smith et al., 2010) may reflect toxicity effects of high metformin doses. Additionally, as lower metformin concentrations extend longevity in male inbred mice (Martin‐Montalvo et al., 2013), but not in heterogeneous mouse populations (Strong et al., 2016), genetic background clearly influences the ability of metformin to impact lifespan in mammals.

Our data identify metformin as an effective intervention for increasing lifespan and healthspan in diverse genetic backgrounds of a *Caenorhabditis* test set. We highlight locomotory health as a critical readout of metformin efficacy, and stress the importance of dose and genetic background in testing for effects. Taken together, consideration of our findings may help in identifying benefits in future metformin studies in model organisms and in human populations.

## 4 EXPERIMENTAL PROCEDURES

### 4.1 Strains

To measure the effect of metformin on lifespan, the following natural isolates were obtained from the *Caenorhabditis* Genetics Center: *C*. *elegans* N2‐PD1073, MY16, JU775; *C*. *briggsae* AF16, ED3092, HK104; and *C*. *tropicalis* JU1373, JU1630, QG834. Worms were maintained at 20°C on NGM agar and fed *E*. *coli* strain OP50‐1. Test subjects were age synchronized by timed egg lays and transferred to media containing FUdR and metformin on day 1 of adulthood. We note that in *C*. *elegans* N2, metformin can increase lifespan regardless of whether or not FUdR is added to the media, suggesting that no synthetic interaction exists between metformin and FUdR (Cabreiro et al., 2013; Onken & Driscoll, 2010) (For full methods, see https://doi.org/10.6084/m9.figshare.9738197.v1).

### 4.2 Genetic diversity

Average nucleotide diversity among the three strains N2, MY16, and JU775 was estimated for diallelic SNPs on regions with 10‐500x coverage using the alignments for each to the *C*. *elegans* reference genome (PRJNA13758, version WS245) from the CeNDR database (release 20200815, https://www.elegansvariation.org). We used samtools v.1.5 and bcftools v.1.5 (Li, 2011), vcftools v.0.1.15 (Danecek et al., 2011), and bedtools v.2.25.0 (Quinlan & Hall, 2010) for variant calling, filtering, and masking (script available: https://github.com/phillips‐lab/CITP_C.elegans_pi).

### 4.3 Interventions

For all experiments, worms were placed on plates containing metformin (1,1‐dimethylbiguanide hydrochloride; Sigma‐Aldrich) or the carrier (water) for control plates on the first day of adulthood, transferred thrice during the first week of adulthood to eliminate progeny and refresh food, and then once weekly to refresh the compound. For survival assays, there were 35–50 worms per plate (technical replicate) and they were observed three times weekly until death. Each lab produced three biological replicates each with three technical replicates at 50 and 70 mM in *C*. *elegans* and two biological replicates at 50 mM in *C*. *briggsae* and *C*. *tropicalis*. In an attempt to find an optimal dose of metformin for the six *C*. *briggsae* and *C*. *tropicalis* strains, each lab performed one survival assay with three technical replicates at each of the following concentrations: 0.1, 1, 5, and 10 mM. (For full methods, see https://doi.org/10.6084/m9.figshare.c.5239976n).

### 4.4 *C*. *elegans* swim test

To determine whether metformin impacts health in nematodes, we utilized the *C*. *elegans* Swim Test (CeleST) to estimate age‐related declines in neuromuscular function (Restif et al., 2014). For these assays, worms were raised as outlined above and moved four at a time to 50 µl pools of 0.1% Tween 20 in M9 on welled slides at the following ages: adult days 6 and 12 in *C*. *elegans* and *tropicalis*, and adult days 8 and 16 in *C*. *briggsae*. Each CeleST recording was performed for 30 s in less than five minutes upon animals entering the liquid environment. In total, for two ages of each strain at 50 mM metformin, 48 animals were measured in each of two biological replicates per lab. (For full methods, see https://doi.org/10.6084/m9.figshare.13377455). The CeleST assay records eight different measures of locomotory ability and body form, which we combined into an adjusted Composite Swimming Score as described (https://doi.org/10.6084/m9.figshare.c.5126579).

## CONFLICT OF INTEREST

The authors declare no competing interest.

## AUTHOR CONTRIBUTIONS

B.O., C.A.S., A.L.C‐H., D.H., P.H., S.G., J.X., E.C., G.H., A.C.F., E.A.C, J.H., D.B., W.T.P., D.I., and M.M performed experiments. B.O., C.A.S., A.L.C‐H., D.H., S.A.B., P.H., E.C., M.G., G.J.L., P.C.P., and M.D. designed experiments and supervised the work. E.J. and E.G.J. performed statistical analyses. B.O. and M.D. wrote the manuscript and analyzed data. C.A.S. generated the figures. A.L.C‐H. and E.G.J. wrote the Supplemental Material. All authors contributed to editing the manuscript.

### OPEN RESEARCH BADGES

This article has earned an Open Data Badge for making publicly available the digitally‐shareable data necessary to reproduce the reported results. The data is available at https://dataverse.harvard.edu/dataverse/CITP/.

## Supporting information

Supplementary Material

Dataset S1

Dataset S2

Dataset S3

Dataset S4

Dataset S5

Dataset S6

Dataset S7

Dataset S8

## Data Availability

The data that support the findings of this study are openly available in: https://dataverse.harvard.edu/dataverse/CITP/

## References

[acel13488-bib-0001] Auton, A. , Abecasis, G. R. , Altshuler, D. M. , Durbin, R. M. , Abecasis, G. R. , Bentley, D. R. , Chakravarti, A. , Clark, A. G. , Donnelly, P. , Eichler, E. E. , Flicek, P. , Gabriel, S. B. , Gibbs, R. A. , Green, E. D. , Hurles, M. E. , Knoppers, B. M. , Korbel, J. O. , Lander, E. S. , Lee, C. , … Abecasis, G. R. (2015). A global reference for human genetic variation. Nature, 526(7571), 68–74. 10.1038/nature15393 26432245 PMC4750478

[acel13488-bib-0002] Bansal, A. , Zhu, L. J. , Yen, K. , & Tissenbaum, H. A. (2015). Uncoupling lifespan and healthspan in Caenorhabditis elegans longevity mutants. Proceedings of the National Academy of Sciences of the United States of America, 112, E277–E286. 25561524 10.1073/pnas.1412192112 PMC4311797

[acel13488-bib-0003] Barzilai, N. , Crandall, J. P. , Kritchevsky, S. B. , & Espeland, M. A. (2016). Metformin as a tool to target aging. Cell Metabolism, 23, 1060–1065. 10.1016/j.cmet.2016.05.011 27304507 PMC5943638

[acel13488-bib-0004] Cabreiro, F. , Au, C. , Leung, K.‐Y. , Vergara‐Irigaray, N. , Cochemé, H. M. , Noori, T. , Weinkove, D. , Schuster, E. , Greene, N. D. E. , & Gems, D. (2013). Metformin retards aging in C. elegans by altering microbial folate and methionine metabolism. Cell, 153, 228–239. 23540700 10.1016/j.cell.2013.02.035 PMC3898468

[acel13488-bib-0005] Chen, J. Ou, Y. , Li, Y. , Hu, S. , Shao, L.‐W. , & Liu, Y. (2017). Metformin extends *C. elegans* lifespan through lysosomal pathway. Elife, 6. 10.7554/eLife.31268 PMC5685485 29027899

[acel13488-bib-0006] Coghlan, A. , & Wolfe, K. H. (2002). Fourfold faster rate of genome rearrangement in nematodes than in Drosophila. Genome Research, 12, 857–867. 10.1101/gr.172702 12045140 PMC1383740

[acel13488-bib-0007] Danecek, P. , Auton, A. , Abecasis, G. , Albers, C. A. , Banks, E. , DePristo, M. A. , Handsaker, R. E. , Lunter, G. , Marth, G. T. , Sherry, S. T. , McVean, G. , & Durbin, R. (2011). The variant call format and VCFtools. Bioinformatics, 27, 2156–2158. 10.1093/bioinformatics/btr330 21653522 PMC3137218

[acel13488-bib-0008] Dugger, S. A. , Platt, A. , & Goldstein, D. B. (2018). Drug development in the era of precision medicine. Nature Reviews Drug Discovery, 17, 183–196. 10.1038/nrd.2017.226 29217837 PMC6287751

[acel13488-bib-0009] Glossmann, H. H. , & Lutz, O. M. D. (2019). Metformin and aging: A review. Gerontology, 65, 581–590. 10.1159/000502257 31522175

[acel13488-bib-0010] Grün, D. , Kirchner, M. , Thierfelder, N. , Stoeckius, M. , Selbach, M. , & Rajewsky, N. (2014). Conservation of mRNA and protein expression during development of *C. elegans* . Cell Reports, 6, 565–577. 24462290 10.1016/j.celrep.2014.01.001

[acel13488-bib-0011] He, L. , & Wondisford, F. E. (2015). Metformin action: concentrations matter. Cell Metabolism, 21, 159–162. 10.1016/j.cmet.2015.01.003 25651170

[acel13488-bib-0012] Hill, R. C. , Egydio de Carvalho, C. , Salogiannis, J. , Schlager, B. , Pilgrim, D. , & Haag, E. S. (2006). Genetic flexibility in the convergent evolution of hermaphroditism in *Caenorhabditis nematodes* . Developmental Cell, 10, 531–538. 10.1016/j.devcel.2006.02.002 16580997

[acel13488-bib-0013] Holden‐Dye, L. , & Walker, R. J. (2014). Anthelmintic drugs and nematicides: studies in *Caenorhabditis elegans* . WormBook 10.1895/wormbook.1.143.2, 1‐29. PMC5402214 25517625

[acel13488-bib-0014] Kaeberlein, M. (2018). How healthy is the healthspan concept? Geroscience, 40, 361–364. 10.1007/s11357-018-0036-9 30084059 PMC6136295

[acel13488-bib-0015] Kiontke, K. C. , Félix, M.‐A. , Ailion, M. , Rockman, M. V. , Braendle, C. , Pénigault, J.‐B. , & Fitch, D. H. A. (2011). A phylogeny and molecular barcodes for Caenorhabditis, with numerous new species from rotting fruits. BMC Evolutionary Biology, 11, 339. 10.1186/1471-2148-11-339 22103856 PMC3277298

[acel13488-bib-0016] Kiontke, K. , & Fitch, D. H. (2005). The phylogenetic relationships of *Caenorhabditis* and other rhabditids. WormBook 10.1895/wormbook.1.11.1, 1‐11. PMC4781183 18050394

[acel13488-bib-0017] Konopka, A. R. , & Miller, B. F. (2019). Taming expectations of metformin as a treatment to extend healthspan. Geroscience, 41, 101–108. 10.1007/s11357-019-00057-3 30746605 PMC6544683

[acel13488-bib-0018] Laranjeiro, R. , Harinath, G. , Burke, D. , Braeckman, B. P. , & Driscoll, M. (2017). Single swim sessions in *C. elegans* induce key features of mammalian exercise. BMC Biology, 15, 30. 10.1186/s12915-017-0368-4 28395669 PMC5385602

[acel13488-bib-0019] Laranjeiro, R. , Harinath, G. , Hewitt, J. E. , Hartman, J. H. , Royal, M. A. , Meyer, J. N. , Vanapalli, S. A. , & Driscoll, M. (2019). Swim exercise in *Caenorhabditis elegans* extends neuromuscular and gut healthspan, enhances learning ability, and protects against neurodegeneration. Proceedings of the National Academy of Sciences of the United States of America, 116, 23829–23839. 31685639 10.1073/pnas.1909210116 PMC6876156

[acel13488-bib-0020] Li, H. (2011). A statistical framework for SNP calling, mutation discovery, association mapping and population genetical parameter estimation from sequencing data. Bioinformatics, 27, 2987–2993. 10.1093/bioinformatics/btr509 21903627 PMC3198575

[acel13488-bib-0021] Lithgow, G. J. , Driscoll, M. , & Phillips, P. (2017). A long journey to reproducible results. Nature, 548, 387–388. 10.1038/548387a 28836615 PMC5762131

[acel13488-bib-0022] Lu, M. R. , Lai, C. K. , Liao, B. Y. , & Tsai, I. J. (2020). Comparative transcriptomics across nematode life cycles reveal gene expression conservation and correlated evolution in adjacent developmental stages. Genome Biology and Evolution, 12, 1019–1030. 10.1093/gbe/evaa110 32467980 PMC7353954

[acel13488-bib-0023] Lucanic, M. , Plummer, W. T. , Chen, E. , Harke, J. , Foulger, A. C. , Onken, B. , Coleman‐Hulbert, A. L. , Dumas, K. J. , Guo, S. , Johnson, E. , Bhaumik, D. , Xue, J. , Crist, A. B. , Presley, M. P. , Harinath, G. , Sedore, C. A. , Chamoli, M. , Kamat, S. , Chen, M. K. , … Phillips, P. C. (2017). Impact of genetic background and experimental reproducibility on identifying chemical compounds with robust longevity effects. Nature Communications, 8, 14256. 10.1038/ncomms14256 PMC5321775 28220799

[acel13488-bib-0024] Ma, P. , & Zemmel, R. (2002). Value of novelty? Nature Reviews Drug Discovery, 1, 571–572. 10.1038/nrd884 12402497

[acel13488-bib-0025] Martin‐Montalvo, A. , Mercken, E. M. , Mitchell, S. J. , Palacios, H. H. , Mote, P. L. , Scheibye‐Knudsen, M. , Gomes, A. P. , Ward, T. M. , Minor, R. K. , Blouin, M.‐J. , Schwab, M. , Pollak, M. , Zhang, Y. , Yu, Y. , Becker, K. G. , Bohr, V. A. , Ingram, D. K. , Sinclair, D. A. , Wolf, N. S. … Cabo, R. (2013). Metformin improves healthspan and lifespan in mice. Nature Communications, 4, 2192. 10.1038/ncomms3192 PMC3736576 23900241

[acel13488-bib-0026] Nam, K. , Munch, K. , Mailund, T. , Nater, A. , Greminger, M. P. , Krützen, M. , Marquès‐Bonet, T. , & Schierup, M. H. (2017). Evidence that the rate of strong selective sweeps increases with population size in the great apes. Proceedings of the National Academy of Sciences of the United States of America, 114, 1613–1618. 10.1073/pnas.1605660114 28137852 PMC5320968

[acel13488-bib-0027] Novelle, M. G. , Ali, A. , Dieguez, C. , Bernier, M. , & de Cabo, R. (2016). Metformin: A hopeful promise in aging research. Cold Spring Harbor Perspectives in Medicine, 6, a025932. 10.1101/cshperspect.a025932 PMC4772077 26931809

[acel13488-bib-0028] Onken, B. , & Driscoll, M. (2010). Metformin induces a dietary restriction‐like state and the oxidative stress response to extend C. elegans Healthspan via AMPK, LKB1, and SKN‐1. PLoS One, 5, e8758. 10.1371/journal.pone.0008758 20090912 PMC2807458

[acel13488-bib-0029] Palliyaguru, D. L. , Moats, J. M. , Di Germanio, C. , Bernier, M. , & de Cabo, R. (2019). Frailty index as a biomarker of lifespan and healthspan: Focus on pharmacological interventions. Mechanisms of Ageing and Development, 180, 42–48. 10.1016/j.mad.2019.03.005 30926563 PMC7307802

[acel13488-bib-0030] Pryor, R. , & Cabreiro, F. (2015). Repurposing metformin: an old drug with new tricks in its binding pockets. The Biochemical Journal, 471, 307–322. 10.1042/BJ20150497 26475449 PMC4613459

[acel13488-bib-0031] Pryor, R. , Norvaisas, P. , Marinos, G. , Best, L. , Thingholm, L. B. , Quintaneiro, L. M. , De Haes, W. , Esser, D. , Waschina, S. , Lujan, C. , Smith, R. L. , Scott, T. A. , Martinez‐Martinez, D. , Woodward, O. , Bryson, K. , Laudes, M. , Lieb, W. , Houtkooper, R. H. , Franke, A. … Cabreiro, F. (2019). Host‐microbe‐drug‐nutrient screen identifies bacterial effectors of metformin therapy. Cell, 178, 1299–1312 e1229. 10.1016/j.cell.2019.08.003 31474368 PMC6736778

[acel13488-bib-0032] Quinlan, A. R. , & Hall, I. M. (2010). BEDTools: a flexible suite of utilities for comparing genomic features. Bioinformatics, 26, 841–842. 10.1093/bioinformatics/btq033 20110278 PMC2832824

[acel13488-bib-0033] Rand, J. B. , & Johnson, C. D. (1995). Genetic pharmacology: interactions between drugs and gene products in Caenorhabditis elegans. Methods in Cell Biology, 48, 187–204. 8531725 10.1016/s0091-679x(08)61388-6

[acel13488-bib-0034] Restif, C. , Ibáñez‐Ventoso, C. , Vora, M. M. , Guo, S. , Metaxas, D. , & Driscoll, M. (2014). CeleST: computer vision software for quantitative analysis of C. elegans swim behavior reveals novel features of locomotion. PLoS Computational Biology, 10, e1003702. 10.1371/journal.pcbi.1003702 25033081 PMC4102393

[acel13488-bib-0035] Sachidanandam, R. , Weissman, D. , Schmidt, S. C. , Kakol, J. M. , Stein, L. D. , Marth, G. , Sherry, S. , Mullikin, J. C. , Mortimore, B. J. , Willey, D. L. , Hunt, S. E. , Cole, C. G. , Coggill, P. C. , Rice, C. M. , Ning, Z. , Rogers, J. , Bentley, D. R. , Kwok, P. Y. , Mardis, E. R. … International SNP Map Working Group (2001). A map of human genome sequence variation containing 1.42 million single nucleotide polymorphisms. Nature, 409, 928–933. 11237013 10.1038/35057149

[acel13488-bib-0036] Slack, C. , Foley, A. , & Partridge, L. (2012). Activation of AMPK by the putative dietary restriction mimetic metformin is insufficient to extend lifespan in Drosophila. PLoS One, 7, e47699. 10.1371/journal.pone.0047699 23077661 PMC3473082

[acel13488-bib-0037] Smith, D. L. Jr , Elam, C. F. , Mattison, J. A. , Lane, M. A. , Roth, G. S. , Ingram, D. K. , & Allison, D. B. (2010). Metformin supplementation and life span in Fischer‐344 rats. The Journals of Gerontology. Series A, Biological Sciences and Medical Sciences, 65, 468–474. 10.1093/gerona/glq033 PMC2854888 20304770

[acel13488-bib-0038] Soubra, R. , Chkeir, A. , & Novella, J. L. (2019). A Systematic Review of Thirty‐One Assessment Tests to Evaluate Mobility in Older Adults. BioMed Research International, 2019, 1354362. 10.1155/2019/1354362 31321227 PMC6610744

[acel13488-bib-0039] Stevens, L. , Félix, M.‐A. , Beltran, T. , Braendle, C. , Caurcel, C. , Fausett, S. , Fitch, D. , Frézal, L. , Gosse, C. , Kaur, T. , Kiontke, K. , Newton, M. D. , Noble, L. M. , Richaud, A. , Rockman, M. V. , Sudhaus, W. , & Blaxter, M. (2019). Comparative genomics of 10 new Caenorhabditis species. Evolution Letters, 3, 217–236. 31007946 10.1002/evl3.110 PMC6457397

[acel13488-bib-0040] Strong, R. , Miller, R. A. , Antebi, A. , Astle, C. M. , Bogue, M. , Denzel, M. S. , Fernandez, E. , Flurkey, K. , Hamilton, K. L. , Lamming, D. W. , Javors, M. A. , de Magalhães, J. P. , Martinez, P. A. , McCord, J. M. , Miller, B. F. , Müller, M. , Nelson, J. F. , Ndukum, J. , Rainger, G. E. … Harrison, D. E. (2016). Longer lifespan in male mice treated with a weakly estrogenic agonist, an antioxidant, an alpha‐glucosidase inhibitor or a Nrf2‐inducer. Aging Cell, 15, 872–884. 27312235 10.1111/acel.12496 PMC5013015

[acel13488-bib-0041] Valencia, W. M. , Palacio, A. , Tamariz, L. , & Florez, H. (2017). Metformin and ageing: improving ageing outcomes beyond glycaemic control. Diabetologia, 60, 1630–1638. 10.1007/s00125-017-4349-5 28770328 PMC5709209

[acel13488-bib-0042] Woodruff, G. C. , Eke, O. , Baird, S. E. , Felix, M. A. , & Haag, E. S. (2010). Insights into species divergence and the evolution of hermaphroditism from fertile interspecies hybrids of Caenorhabditis nematodes. Genetics, 186, 997–1012. 10.1534/genetics.110.120550 20823339 PMC2975280

[acel13488-bib-0043] Wu, L. , Zhou, B. , Oshiro‐Rapley, N. , Li, M. , Paulo, J. A. , Webster, C. M. , Mou, F. , Kacergis, M. C. , Talkowski, M. E. , Carr, C. E. , Gygi, S. P. , Zheng, B. , & Soukas, A. A. (2016). An ancient, unified mechanism for metformin growth inhibition in *C. elegans* and cancer. Cell, 167, 1705–1718 e1713. 27984722 10.1016/j.cell.2016.11.055 PMC5390486

