# Supplementary material for: Metformin treatment of diverse Caenorhabditis species reveals the importance of genetic background in longevity and healthspan extension outcomes
Source: Aging Cell. 2021 Nov 27;21(1):e13488. doi: 10.1111/acel.13488 (PMC8761014; doi:10.1111/acel.13488)
Supplement: Supplementary file 1 — Supplementary Material [file ACEL-21-e13488-s006.docx]

**Supplementary Information Text**

**Details on Datasets**

The main statistical analyses for all lifespan data are separated by strain. Each strain is analyzed using a restricted maximum likelihood general linear model and a Cox Proportional Hazards model. When particular concentrations for a strain are considered, only the control records from those same trials are included. For each lifespan model of the age at death, the predictor variable is the concentration, and the nested random effects are lab, experimenter, trial start date, and plate ID. Hierarchical mixed effects models are chosen to handle the sources of variation from among and within labs. This allows us to pool the experiments together by strain while still considering the impact from different biological trials and technical replicates. For lifespan data here and in general, most trial variance is between biological trials, not within technical replicates.

From each model, multiple comparisons of various concentrations of metformin versus the control for each strain are in Dataset S2 and variance components estimates of the random effects are in Dataset S3. Since each strain is analyzed separately, while a given trial date includes various strains, those strain-trial date combinations are independent. While species are separated for certain figures, all strains are listed together in Datasets S2 and S3.

Additional summary information of all lifespan data are included in Dataset S4. Three main summary tables for each strain-concentration, strain-concentration-trial, and strain-concentration-trial-plate display the Kaplan-Meier medians, means, age at 90% failures, and maximum ages. A final table displays one-way ANOVA results of the plate medians for each strain-concentration, focusing on the trial variance. It is evident that most trial variance is between biological trials, not within technical replicates.

**Dataset S1 (separate file).** Variance components estimates and significance tests for the adjusted swimming score, analyzed separately for each strain. Linear discriminant analysis output for each strain. Includes R packages *lme4* (3), *multcomp* (5), *car* (1), and *MASS* (4).

**Dataset S2 (separate file).** Significance tests for metformin interventions effects on longevity. Includes R package *multcomp* (5). The variance components estimates of these same models are in Dataset S3.

**Dataset S3 (separate file).** Variance components estimates for longevity, analyzed separately for each strain, at all concentrations combined. Includes R packages *lme4* (3) and *coxme* (2).

**Dataset S4 (separate file).** Summary of manual lifespan data under metformin treatment at concentrations from 0.1 to 70 mM. Includes R packages *stats* (6) and *survival* (7).

**Dataset S5 (separate file).** All raw survival data.

**Dataset S6 (separate file).** All raw CeleST data plus linear discriminant analysis results and composite swimming scores.

**Dataset S7 (separate file).** All R scripts for survival data under metformin treatment or other anti-diabetes compounds.

Dataset S8 (separate file). All R scripts for CeleST data, including creating the adjusted swimming score.

All Datasets available at https://dataverse.harvard.edu/dataverse/CITP/

The CeleST Parameters of Locomotion and Body Posture

We weighted eight different measurement values to generate a composite swimming score. The parameters (as described in Restif et al. 2014) are:

Wave Initiation Rate, the number of body waves initiated from either the head or tail per minute.

Body Wave Number, the number of waves in transit through the body at a point in time.

Asymmetry, a measure of how balanced the swim posture is per stroke.

Stretch, which reports on whether body bends are deep or flat and how much “stretching” effort occurs in a stroke.

Curling, which measures the relative percentage of time that an animal spends bent around so far that it overlaps with itself.

Travel Speed, the distance that an animal travels during a defined time.

Brush Stroke, the number of pixels covered by the body of an animal in a single stroke.

Activity Index, the number of pixels that are covered by the body during the time it takes an animal to do two strokes, a measure of how vigorously the animal bends over time.

The eight measures of posture and swimming ability are used to generate an adjusted swimming score for each individual worm. A restricted maximum likelihood general linear model is created for the adjusted swimming score, similar to the lifespan models. The predictor variables are the age and compound or control, including an interaction term. The nested random effects are lab, experimenter, trial date, and video.

All output from the linear model and other CeleST analyses are in tables in Dataset S1 and can be matched to the appropriate R scripts listed in Dataset S8. This includes a Type-III ANOVA on the age by compound interaction for each strain. The result of interest is the significance of the interaction effect, and whether or not the size of a compound’s impact is significantly impacted by age. Similar to Dataset S4, a summary table of the medians for each strain-compound-age-trial-video is included, along with one-way ANOVA results of those medians for each strain-compound-age.

Metformin Stands Out as A Health-Promoting Anti-Diabetes Medication

To consider the possibility that anti-diabetes interventions in general might improve health in *Caenorhabditis* strains, we examined potential lifespan effects of six additional anti-diabetes medications in limited trials (one lab, one biological replicate, three technical replicates for each) (Fig. S3). Of these, five interventions conferred modest effects, were toxic, or did not impact lifespan. One, bromocriptine, demonstrated a complex impact on *C. elegans* and *C. briggsae* with lower doses having no effect and high doses being toxic, while an intermediate dose confers beneficial lifespan effects in *C. elegans* only. In *C. tropicalis*, the highest tested bromocriptine dose (toxic in the other two species) also significantly extended lifespan. Overall, bromocriptine promoted longevity in two *Caenorhabditis* species, although dose is critical and toxicity is a concern. Metformin, with positive lifespan/healthspan impact in three *C. elegans* strains at 50 and 70 mM concentrations and positive healthspan impact at 50 mM in *C. briggsae* has robust effects that appear considerably stronger than those of bromocriptine (in *C. elegans* N2, 50 µM bromocriptine increases median survival by 12%, the only beneficial dose). Additional bromocriptine replicates in all strains will be necessary for detailed comparison and reproducibility, but the primary finding is that metformin appears specific in its robust capacity to extend healthspan in *Caenorhabditis elegans* strains and this capacity is not generally shared with other diabetes interventions.

**Fig. S1.** Kaplan-Meier curves showing variation within labs across biological and technical replicates for the 50 mM metformin dataset used to generate the estimates of sources of variance in supplemental table 1. Each plot is a lab by strain combination, where each curve represents one technical replicate plate of approximately 40 individuals. The curves are colored by treatment (black- control, teal- 50 mM metformin), with the stroke pattern corresponding to the trial within a given lab. In general. each technical replicate within a trial and lab was conducted by a different experimenter.

Fig. S2. Metformin increases maximum lifespan in all *C. elegans* strains. The 90% cutoff calculated from the Kaplan-Meier survival estimates are shown for *C. elegans* strains N2, JU775, and MY16 on control (vehicle only), and metformin treated (50 and 70 mM) plates. Each point represents an individual trial plate, and the error bars represent the mean of the 90% quartile +/- the standard error of the mean. Replicates were conducted at the three CITP testing sites (blue square- Buck, red diamond- Rutgers, dark green circle- Oregon). Asterisks represent *p-*values from the CPH model such that *****p*<0.0001, ****p*<0.001, ***p*<0.01, and **p*<0.05.

Fig. S3. Six anti-diabetic compounds have little to no positive effect on lifespan in three *Caenorhabditis* species. Kaplan-Meier survival curves are shown for *C. elegans* strain N2, *C. briggsae* strain AF16, and *C. tropicalis* strain JU1630 exposed to various concentrations of (a) bromocriptine mesylate, (b) dapagliflozin, (c) sitagliptin phosphate, (d) nateglinide, (e) pioglitazone hydrochloride, or (f) glipizide starting on day one of adulthood. Data shown are from a single trial completed at one CITP testing site (Oregon), with curves consisting of multiple plate replicates. Asterisks represent *p*-values from the CPH model such that *****p*<0.0001, ****p*<0.001, ***p*<0.01, and **p*<0.05.

**Table S1.** Comparison of reproducibility of longevity estimates for manual lifespan assays with pharmacological intervention on 50 mM metformin (plus controls) for nine strains across three species. (See Table S2 for full variance components). Results for other concentrations are excluded from this analysis. Results for CeleST on 50 mM metformin are the average of the variance components estimates analyzed separately for each individual measure (Dataset S1).

| **Source of Variation** | **Survival Assays** | **CeleST Measure Average** |
| --- | --- | --- |
| **Genetic Variation** | **19.2** | **19.1** |
| Among species | 5.6 | 12.6 |
| Among strains w/in species | 8.7 | 1.6 |
| Species x age |  | 2.7 |
| Species x compound | 4.9 | 0.0 |
| Species x age x compound |  | 0.2 |
| Strain x age |  | 0.6 |
| Strain x compound | 0.1 | 0.2 |
| Strain x age x compound |  | 1.1 |
| **Reproducibility Among Labs** | **5.1** | **8.3** |
| Among labs | 0.0 | 4.5 |
| Lab x species | 3.6 | 0.8 |
| Lab x strain | 0.9 | 1.8 |
| Lab x age |  | 0.6 |
| Lab x compound | 0.6 | 0.1 |
| Lab x age x compound |  | 0.7 |
| **Reproducibility Within Labs** | **12.0** | **9.4** |
| Among experimenters | 0.7 | 0.9 |
| Among trials w/in experimenters | 4.4 | 3.0 |
| Among plates w/in trials | 7.0 |  |
| Among videos w/in trials |  | 5.4 |
| **Individual Variation** | **63.6** | **63.2** |
|  |  |  |
| **Total** | **100.0** | **100.0** |
|  |  |  |
| Total number of observations | 16,432 | 9,104 |

**Table S2.** Partitioning of variation for longevity across genetic background and experimental replicates within and among labs at 50 mM metformin. Variance components were estimated as a randomized block design using a restricted maximum likelihood (REML) general linear model using the *lme4* (3) package of R. All factors were treated as random effects. Tests involving the fixed compound effects are reported in Dataset S2.

| **Source** | **Variance Component** | | **Lower**  **95% CI** | | **Upper**  **95% CI** | | **Percent**  **of Total** | |
| --- | --- | --- | --- | --- | --- | --- | --- | --- |
| Species | 4.09E+00 | 0.00E+00 | | 7.19E+00 | | 5.6 | |  |
| Strain*Species | 6.37E+00 | 1.48E+00 | | 5.01E+00 | | 8.7 | |  |
| Compound*Species | 3.59E+00 | 7.56E-01 | | 4.28E+00 | | 4.9 | |  |
| Compound*Strain | 1.02E-01 | 0.00E+00 | | 1.10E+00 | | 0.1 | |  |
| Lab | 0.00E+00 | 0.00E+00 | | 3.64E+00 | | 0.0 | |  |
| Lab*Species | 2.66E+00 | 5.61E-01 | | 3.67E+00 | | 3.6 | |  |
| Lab*Strain | 6.76E-01 | 2.73E-01 | | 1.58E+00 | | 0.9 | |  |
| Compound*Lab | 4.49E-01 | 1.55E-01 | | 2.00E+00 | | 0.6 | |  |
| Experimenter*Lab | 4.95E-01 | 0.00E+00 | | 1.77E+00 | | 0.7 | |  |
| Trial[Experimenter, Lab] | 3.22E+00 | 1.29E+00 | | 2.44E+00 | | 4.4 | |  |
| Plate[Trial, Experimenter, Lab] | 5.14E+00 | 2.05E+00 | | 2.50E+00 | | 7.0 | |  |
| Residual | 4.68E+01 | 6.77E+00 | | 6.92E+00 | | 63.6 | |  |
| Total | 7.36E+01 |  | |  | | 100.0 | |  |

**SI References**

Restif, C, C Ibanez-Ventoso, M Vora, S Guo, D. Metaxas and M Driscoll (2014). CeleST: computer vision software for quantitative analysis of C. elegans swim behavior reveals novel features of locomotion. PLoS Comput Biol. 10(7):e1003702.

R Packages

**car:** Fox, J and S Weisberg (2019). A {R} Companion to Applied Regression, Third Edition. Thousand Oaks CA: Sage. R package version 3.0-10. <https://socialsciences.mcmaster.ca/jfox/Books/Companion/>. <https://cran.r-project.org/web/packages/car>.

**coxme:** Therneau, T (2018). coxme: Mixed Effects Cox Models. R package version 2.2-16. <https://CRAN.R-project.org/package=coxme>.

**lme4:** Bates D, M Maechler, B Bolker, and S Walker (2015). Fitting Linear Mixed-Effects Models Using lme4. Journal of Statistical Software, 67(1), 1-48. R package version 1.1-23. doi:10.18637/jss.v067.i01. <https://cran.r-project.org/web/packages/lme4>.

**MASS:** Venables, W N and B D Ripley (2002). Modern Applied Statistics with S. Fourth Edition. Springer, New York. ISBN 0-387-95457-0. R Package Version 7.3-51. <http://www.stats.ox.ac.uk/pub/MASS4/>.

**multcomp:** Hothorn T, F Bretz, and P Westfall (2008). Simultaneous Inference in General Parametric Models. Biometrical Journal 50(3), 346-363. R package version 1.4-12. <https://CRAN.R-project.org/package=multcomp>

**stats:** R Core Team (2019). R: A language and environment for statistical computing. R Foundation for Statistical Computing, Vienna, Austria. R package version 3.6-2. <https://www.R-project.org/>.

**survival:** Therneau T (2015). _A Package for Survival Analysis in S_. R Package version 3.2.7. <https://CRAN.R-project.org/package=survival>.
